# ﻿Four new earthworm species of the genera *Amynthas* and *Metaphire* (Oligochaeta, Megascolecidae) from Hunan and Anhui provinces, China

**DOI:** 10.3897/zookeys.1210.125963

**Published:** 2024-08-26

**Authors:** Qing Jin, Jiali Li, Jibao Jiang, Jiangping Qiu

**Affiliations:** 1 School of Agriculture and Biology, Shanghai Jiao Tong University, Shanghai, China Shanghai Jiao Tong University Shanghai China; 2 Shanghai Urban Forest Research Station, State Forestry Administration, Shanghai, China Shanghai Urban Forest Research Station, State Forestry Administration Shanghai China

**Keywords:** Barcode, COI gene, Megascolecidae, new species, Oligochaeta, taxonomy

## Abstract

This paper describes four new species earthworms from Hunan and Anhui provinces, China, *Amynthasxiangtanensis* Qiu & Jin, **sp. nov.**, *Amynthastaoyuanensis* Qiu & Jin, **sp. nov.**, *Amynthasxuanchengensis* Jin & Li, **sp. nov.** and *Metaphiredonganensis* Jin & Jiang, **sp. nov.***Amynthasxiangtanensis***sp. nov.**, and *A.taoyuanensis***sp. nov.** belong to the *Amynthascorticis* group. Both have four pairs of intersegmental spermathecal pores in 5/6–8/9; male pores in segment XVIII, separated by 1/3 of body circumference, each on top of a slightly raised porophore, surrounded by several tiny genital papillae. *Amynthastaoyuanensis***sp. nov.** prostate glands are degenerated. *Amynthasxuanchengensis***sp. nov.** belongs to the *Amynthasmorrisi* group, it has two pairs of spermathecal pores in 5/6 and 6/7; male pores in XVIII, separated by 1/3 of body circumference, each on top of a slightly raised, circular porophore. *Metaphiredonganensis***sp. nov.** belongs to the *Metaphirehoulleti* group. It has three pairs of spermathecal pores in 6/7–8/9; male pores in XVIII, separated by 1/3 of body circumference, each on the bottom center of the longitudinal copulatory chamber.

## ﻿Introduction

Earthworms belonging to the family Megascolecidae are the most important and widely distributed in China. Hunan Province (24°38'–30°08'N, 108°47'–114°15'E) is located in the south of China and has a subtropical monsoon climate. Anhui Province (29°41'–34°38'N, 114°54'–119°37'E) is located in the east of China, a transitional region between the warm temperate zone and the subtropical zone. Both provinces are among the most abundant biological diversity areas in China, yet only a few earthworm species have been reported there. Previously, only five (*Amynthasmoniliatusmoniliatus* (Chen, 1946), *A.triastriatustriastriatus* (Chen, 1946), *Metaphirebiforatum* Tan & Zhong, 1987, *M.bifoliolare* Tan & Zhong, 1987 and *M.hunanensis* Tan & Zhong, 1986) and eight (*A.carnosuscarnosus* (Goto & Hatai, 1899), *A.corticis* (Kinberg, 1867), *A.hupeiensis* (Michaelsen, 1895), *A.lot*i (Chen & Hsu, 1975), *A.pectieniferus* (Michaelsen, 1931), *A.robustus* (Perrier, 1872), *M.guillelmi* (Michaelsen, 1895) and *M.tschiliensistschiliensis* (Michaelsen, 1928)) Megascolecidae species have been recorded from Hunan and Anhui, respectively (Chen 1946, 1959; Chen and Hsu 1977; Zeng et al. 1982; Tan and Zhong 1986, 1987).

**Figure 1.**
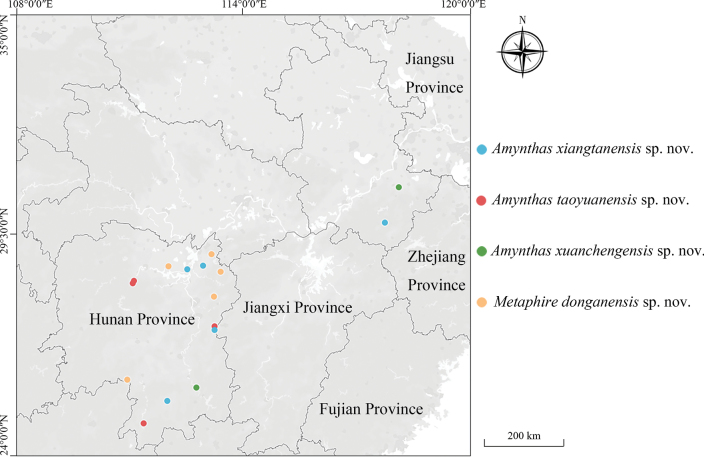
Distribution of four species in Hunan Province and Anhui provinces, China.

In 2015, 2016, and 2019, we investigated earthworm diversity in the two provinces and more than 31 and 24 Megascolecidae species were recorded from Hunan and Anhui, respectively. In this paper, we describe three new species of the genus *Amynthas* and one new species of the genus *Metaphire* found in those surveys (Fig. 1). Among them, *A.xiangtanensis* sp. nov. and *A.taoyuanensis* sp. nov. belong to the *A.corticis* group with intersegmental spermathecal pores in 5/6–8/9, which is widely distributed in China (such as Hainan, Yunnan, and Guangxi provinces) (Sun et al. 2012, 2018, 2021; Dong et al. 2019; Yuan et al. 2019). *Amynthasxuanchengensis* sp. nov. belongs to the *A.morrisi* group with intersegmental spermathecal pores in 5/6 and 6/7, which is widely distributed in China including Hainan, Yunnan, and Guangdong provinces (Zhao et al. 2013; Jiang et al. 2015; Sun et al. 2015, 2021). *Metaphiredonganensis* sp. nov. belongs to the *M.houlleti* group, with intersegmental spermathecal pores in 6/7–8/9, which is also widely distributed in China including Jiangxi, Gansu, and Fujian provinces (Feng 1984; Sun et al. 2018). DNA barcodes of the four new species are provided (PP497092–PP497100) in this paper.

## ﻿Materials and methods

The earthworms were collected in 2015, 2016, and 2019. Specimens were anaesthetized in a 10% ethanol solution and preserved in a 95% ethanol solution. Holotypes and paratypes are deposited in the Shanghai Natural History Museum.

DNA was extracted from several specimens of *A.xiangtanensis* sp. nov., *A.taoyuanensis* sp. nov., *A.xuanchengensis* sp. nov., *M.donganensis* sp. nov. by using the E.Z.N.A. Mollusc DNA Kit (Omega Bio-tek, Norcross, GA, USA). The gene cytochrome c oxidase subunit I (COI) was amplified by polymerase chain reaction (PCR). The PCR amplification mixture (50 μL total) consisted of 1 μL of DNA template, 2 μL of each primer, 35.4 μL double-distilled H_2_O, and 9.6 μL Trans Taq^TM^ Polymerase High Fidelity containing 0.6 μL TransTaqTM HiFi DNA polymerase, 4 μL 2.5 mM dNTPs and 5 μL 10 × TransTaq^TM^ HiFi Buffer I. Primers used in the research were COI,5’-GGTCAACAAATCATAAAGATATTGG-3’ and 5’-TAAACTTCAGGGTGACCAAAAAATCA-3’ (Folmer et al. 1994). The PCR was carried out as follows: 5 min at 94 °C followed by 32 cycles 94 °C for 30 s, 50 °C for 30 s and 72 °C for 60 s, with an extension of 10 min at 72 °C. Sequencing was performed in the Beijing Genomics Institute (Shanghai, China). COI sequences of the new species were submitted to the NCBI GenBank databases under the accession numbers provided in Table 1, and sequences of other similar known species were retrieved from GenBank (Table 1). All the DNA sequences were aligned using ClustalX 2.0 (Thompson et al. 1997). The genetic pairwise distances between these species were calculated using the Kimura two-parameter model (Kimura 1980) in MEGAX with 1000 bootstrap replicates (Kumar et al. 2018).

**Table 1.** Specimens with molecular data used in this study. Some species have no molecular data in GenBank. Abbreviations, HT holotype, PT paratype.

Species	Species number	GenBank acc. no.
*Amynthasxiangtanensis* sp. nov. (HT)	P1CJHUSH190510083 N1-05A	PP497097
*Amynthasxiangtanensis* sp. nov. (PT)	P1CJHUSH190519808 N5-02	PP497098
*Amynthasxiangtanensis* sp. nov. (PT)	P1CJHUSH190526781 N5-01	PP497099
*Amynthastaoyuanensis* sp. nov. (HT)	P1CJHUSH190517069 N8-01A	PP497095
*Amynthastaoyuanensis* sp. nov. (PT)	P1CJHUSH190526781 R8-04	PP497096
*Amynthasxuanchengensis* sp. nov. (PT)	P1CJHUSH190521800 Q6-03	PP497100
*Metaphiredonganensis* sp. nov. (PT)	P1CJHUSH190511779 N9-03	PP497092
*Metaphiredonganensis* sp. nov. (PT)	P1CJHUSH190512778 N11-01	PP497094
*Metaphiredonganensis* sp. nov. (PT)	P1CJHUSH190512096 Q3-04	PP497093
*Amynthascorticis* (Kingberg, 1867)	HN201035-02	KF205966
*Amynthasmaximus* Qiu & Dong, 2019	GX201304-01	MG450707
*Amynthastortuosus* Qiu & Dong, 2019	GX201306-06	MG450708
*Amynthasstricosus* Qiu & Sun, 2012	HN201104-04	JX315345
*Amynthashomosetus* (Chen, 1938)	-	No data in GenBank
*Amynthasgenitalis* Qiu & Sun, 2012	-	No data in GenBank
*Amynthasrecavus* Yuan & Jiang, 2019	YN201109-09	KF205473
*Amynthasendophilus* Zhao & Qiu, 2013	HN201011-03	KF240560
*Amynthasfucatus* Zhao & Qiu, 2013	HN201114-01	KF151185
*Amynthasinfuscuatus* Jiang & Sun, 2015	-	No data in GenBank
*Amynthaszonarius* Sun & Qiu, 2015	HN201114-06	JQ982486
*Amynthasbaikmudongensis* Hong, 2017	-	No data in GenBank
*Metaphirevulgarisagricola* (Chen,1930)	-	No data in GenBank
*Metaphiretschiliensislanzhouensis* (Feng, 1984)	-	No data in GenBank
*Metaphireviridis* Feng & Ma, 1987	-	No data in GenBank
*Metaphireptychosiphona* Qiu & Zhong, 1993	-	No data in GenBank
*Metaphiresanmingensis* Sun & Jiang, 2018	FJ201008-02	KY774380

## ﻿Taxonomy

### ﻿Family Megascolecidae Rosa, 1891

**Genus *Amynthas* Kinberg, 1867**

#### ﻿ Amynthas xiangtanensis

Taxon classificationAnimaliaOligochaetaMegascolecidae

Qiu & Jin sp. nov.

535BD2BD-982E-50CC-8BE7-D696AADC90C1

https://zoobank.org/63FF5853-828D-40A1-A933-8A108DEF9196

Fig. 2

##### Material examined.

***Holotype*.** • 1 clitellate (P1CJHUSH190510083 N1-05A), China, Hunan Province, Xiangtan City (27.98312°N, 112.81616°E), 47 m elevation, brownish yellow soil under shrub in farmland, 10 May 2019, JB Jiang, JL Li and BY Yin. ***Paratypes*.** 10 clitellates in total • 2 clitellates (P1CJHUSH190510083 N1-05B), China, Hunan Province, Xiangtan City (27.98312°N, 112.81616°E), 47 m elevation, brownish yellow soil under shrub in farmland, 10 May 2019, JB Jiang, JL Li and BY Yin • 2 clitellates (P1CJHUSH190519808 N5-02), China, Hunan Province, Yongzhou City (25.83566°N, 112.27331°E), 163 m elevation, brown soil under weeds in field, 19 May 2019, JB Jiang, JL Li and Y Wang • 2 clitellates (P1CJHUSH190526781 N5-01), China, Hunan Province, Liling City (27.54671°N, 113.54837°E), 74 m elevation, brown soil under weeds in farmland, 26 May 2019, JB Jiang, JL Li and Y Wang • 2 clitellates (P1CJHUSH190514791 N13-01), China, Hunan Province, Yueyang City (29.07035°N, 113.23038°E), 57 m elevation, yellow soil under vegetable field in farmland, 14 May 2019, Y Dong, YF Qin and YZ Wu • 2 clitellates (AH201612-02), China, Anhui Province, Huangshan City (30.58531°N, 117.87033°E), 506 m elevation, brown soil under vegetable field in farmland, 8 May 2016, JB Jiang, J Sun, Y Dong and Y Zheng.

##### Diagnosis.

Size medium to large. Spermathecal pores in 5/6–8/9, separated by 1/3 of body circumference. Male pores in XVIII, separated by 1/3 of body circumference, each on the top of a slightly raised, circular porophore. Spermathecae four pairs in VI–IX, ampulla heart-shaped, duct thick and ~ 1/4 of ampulla. Diverticulum is ~ 2/3 of main pouch (duct and ampulla together), terminal 1/2 dilated into rod-shaped seminal chamber. Intestinal caeca are simple. Prostate glands are well developed.

##### External characters.

**Figure 2.**
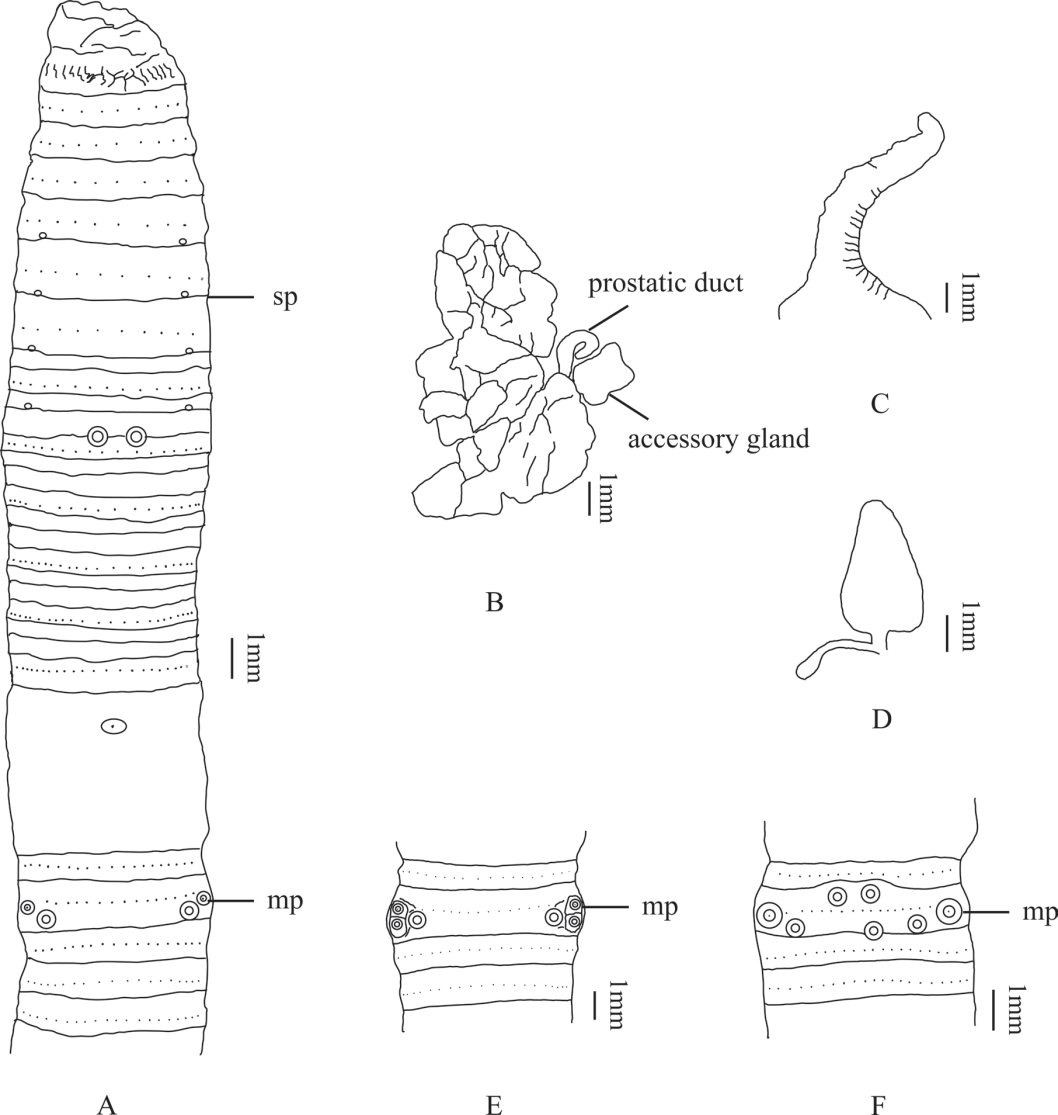
*A.xiangtanensis* sp. nov. **A** ventral view showing spermathecal pores, and male pores **B** prostate glands **C** intestinal caeca **D** spermathecae **E** male pores of paratypes (P1CJHUSH190519808 N5-02) **F** male pores of paratypes (P1CJHUSH190514791 N13-01). Abbreviations: sp, spermathecal pores; mp, male pores.

Yellowish brown dorsal pigmentation, pale yellowish brown ventral pigmentation. Dimensions 110–184 mm by 5.0–7.0 mm at clitellum, segments 111–133. Annulus present on VIII–XIII. The dorsal midline is clearly visible and purplish brown. First dorsal pore of all examined individuals in 9/10. Prostomium 1/2 epilobous. Clitellum annular, taupe, in XIV– XVI, swollen, setae invisible externally, but dorsal pores visible on clitellum. Setae numbering 16–28 at III, 26–32 at V, 40–46 at VIII, 46–54 at XX, 54–58 at XXV; 14–16 between male pores; 8–12 (V), 10–12 (VI), 12–14 (VII) and 14–17 (VIII) between spermathecal pores; setal formula, aa = 1.0–1.2ab, zz = 1.4–2.0zy. Male pores one pair in XVIII, separated by 1/3 of body circumference, each on the top of a slightly raised, circular porophore. A pair of oval medium-sized flat-topped papillae on XVIII, after the setae ring near male pores, the interval is ~ 1/4 of body circumference (Fig. 2A). Sometimes another pair of similar papillae present after male pores, the interval is ~ 1/3 of body circumference (specimen P1CJHUSH190519808 N5-02) (Fig. 2E); another pair similar papillae present on the ventrum of XVIII, but near to the ventral line (specimen P1CJHUSH190514791 N13-01) (Fig. 2F). Female pore single in XIV, oval, milky white. Spermathecal pores four pairs in 5/6–8/9, ventral, small eye-like, milky white porophore in center, separated by 1/3 of body circumference. A pair of oval medium-sized flat-topped papillae before the setae ring on the ventrum of IX, the interval of two papillae separated by ~ 1/9 of body circumference (Fig. 2A). Sometimes another pair of similar papillae present on the same position of VIII (specimen P1CJHUSH190526781 N5-01).

##### Internal characters.

Septa 5/6–7/8 thick and muscular, 10/11–12/13 slightly thickened, 8/9 and 9/10 absent. Gizzard long bucket-shaped, in IX–X. Intestine enlarged distinctly from XV. Intestinal caeca paired in XXVII, extending anteriorly to XXIII, simple, smooth on both sides or sometimes weakly constricted on ventral margin (Fig. 2C). Four esophageal hearts in X–XIII, the latter three are more developed than the first pair. Male sexual system holandric, testis sacs two pairs in X and XI, well developed, left and right lobes separated on the ventral side. Seminal vesicles two pairs in XI and XII, well developed, left and right lobes separated on the ventral side. Prostate glands well developed, inserting in XVIII and extending to XIV and XXI, coarsely lobate, prostatic duct U-shaped, slightly thicker at the distal part, a large lumpy accessory gland beside the prostatic duct (Fig. 2B). Spermathecae four pairs in VI–IX, ampulla heart-shaped, ~ 2.5–4.5 mm long in holotype; ampulla duct is thick and ~ 1/4 of ampulla. Diverticulum is ~ 2/3 of main pouch (duct and ampulla together), slender, terminal 1/2 dilated into rod-shaped seminal chamber. A pair of large lumpy accessory glands present on the ventrum of IX, corresponding to the position of papillae (Fig. 2D). Sometimes another pair of large lumpy accessory glands present on the ventrum of VIII, corresponding to the position of papillae.

##### Etymology.

The species is named after its type locality.

##### Remarks.

*Amynthasxiangtanensis* sp. nov., with four pairs of spermathecal pores in 5/6–8/9, keys to the *Amynthascorticis* group (Sims and Easton 1972) which consists of 111 species (Nguyen et al. 2020a, 2020b; Sun et al. 2021; Li et al. 2024); it is similar to *A.taoyuanensis* sp. nov. in setae number, spermathecal pores, male pores, and simple intestinal caeca (Table 2). However, *A.xiangtanensis* sp. nov. (110–184*5.0–7.0, yellowish brown dorsum and pale yellowish brown ventrum) is easily distinguished from *A.taoyuanensis* sp. nov. (length 41–120*3.5–4.5, colorless) by its larger body size and pigmentation. In addition, the first dorsal pore of *A.xiangtanensis* sp. nov. is in 9/10, but 10/11 in *A.taoyuanensis* sp. nov.; clitellum in XIV–XVI of *A.xiangtanensis* sp. nov., while XIV–2/3XVI in *A.taoyuanensis* sp. nov.; paired papillae before setae in VIII or IX in *A.xiangtanensis* sp. nov., but paired papillae after setae in VI, VII or VIII in *A.taoyuanensis* sp. nov.; two or more papillae near male pore of *A.xiangtanensis* sp. nov., whereas two pairs or more in XVI, XVII, XVIII or XIX of *A.taoyuanensis* sp. nov.; prostate gland well developed in *A.xiangtanensis* sp. nov., nevertheless prostate glands degenerated in *A.taoyuanensis* sp. nov.; spermathecae of *A.xiangtanensis* sp. nov. (~ 2.5–4.5 mm long, ampulla heart-shaped, duct ~ 1/4 of ampulla) are different from *A.taoyuanensis* sp. nov. (~ 0.6–1.8 mm long, ampulla oval-shaped, duct is thick and ~ 1/2 of ampulla) by size and shape; diverticulum terminal 1/2 dilated into rod-shaped seminal chamber in *A.xiangtanensis* sp. nov., but terminal 3/4 dilated into bag-shaped seminal chamber in *A.taoyuanensis* sp. nov.

**Table 2.** A comparison of characters of *A.xiangtanensis* sp. nov., *A.taoyuanensis* sp. nov., and similar species of the *Amynthascorticis* group. Abbreviations: sp, spermathecal pores, mp, male pores.

Character	*A.xiangtanensis*	*A.taoyuanensis*	*A.corticis*	*A.maximus*	*A.tortuosus*	*A.stricosus*	*A.homosetus*	*A.genitalis*	*A.recavus*
Body size (mm)	110–184*5.0–7.0	41–120*3.5–4.5	45–170*3.0–6.0	145–170*5.8–6.2	55–86*2.5–2.8	72–97*2–2.8	116*5.2	83–97*2.3–2.5	58-64*2.1–2.3
Pigment dorsum	Yellowish brown	None	Greenish brown	Pale purple-brown to brown	Purple-brown to pale purple-brown	None	Dark chocolate to grey	None	Pink to pale brown
Pigment ventrum	Pale yellowish brown	None	None	None to yellowish	Pale purple-brown to None	None	Grey	None	None
First dorsal pore	9/10	10/11	11/12	13/14	13/14	11/12 or 12/13	12/13	12/13	12/13
Clitellum	XIV–XVI	XIV– 2/3XVI	XIV–XVI	2/5XIV–XVI	XIV–XVI	XIV–XVI	XIV–XVI	XIV–XVI	XIV–XVI
Setae numbering	16–28/III, 40–46/VIII, 46–54/XX	32–42/III, 44–50/VIII, 50–58/XX	36–40/VII, 40–46/XXV	33–38/III, 29–33/VIII, 18–22/XX	24–26/III, 34–36/VIII, 32–36/XX	30–54/III, 62–72/VIII, 40–70/XX	44/VIII	30–36/III, 32–36/VIII, 38–46/XX	21–22/III, 34–36/VIII, 34–36/XX
Setae number between sp	14–17 (VIII)	19–22 (VIII)	12 (VIII)	18–22 (VIII)	12–13 (VIII)	23–29 (VIII)	-	8–11 (VIII)	12 (VIII)
Setae number between mp	14–16 (XVIII)	13–14 (XVIII)	10–14 (XVIII)	9–13 (XVIII)	8–9 (XVIII)	10–12 (XVIII)	9(XVIII)	11–12 (XVIII)	9–10 (XVIII)
Ventral distance of sp	1/3C	1/3C	1/3C	1/3C	1/4C	2/5C	1/4C	1/3C	2/5C
Papillae within sp region	Paired before setae in VIII or IX	Paired after setae in VI, VII or VIII	Paired before or after setae near sp	Two pairs after setae in VII and VIII	Four pairs after setae in VI–IX	None	Invisible	None	None
Papillae within mp region	Two or more near male pore	Two paired or more in XVI, XVII, XVIII, or XIX	One or more papillae near male pore	Paired before setae medial of male pore	Paired medial of male pore	Single or paired in XVII, XIX, and XX	Invisible	4 paired in XVII, XVIII and XIX	Paired in XVII, 3 papillae in XIX
Prostate glands	Well developed with accessory gland	Degenerated with accessory glands	Developed	Underdeveloped	Developed	Developed,	Developed	Developed	Developed
Diverticulum	2/3 of main pouch, terminal 1/2 dilated into rod-shaped seminal chamber	2/3 of main pouch, terminal 2/3 dilated into bag-shaped seminal chamber	Shorter than main pouch and terminal dilated into round or elongate oval seminal chamber	Shorter, lightly twist in middle, terminal 2/5, dilated into rod-shaped seminal chamber	Shorter, terminal 4/5 dilated into S-shaped twisted seminal chamber	As long as main pouch, slender, terminal 2/5 dilated into a band shaped chamber	Shorter, terminal dilated into rod-shaped seminal chamber	Longer than main pouch, terminal 0.29 dilated into rod-shaped seminal chamber	~ 3/5 of main pouch, terminal 1/4 dilated into ovoid-shaped seminal chamber
Accessory glands	Paired on VIII or IX	Two paired on VII or VIII	Bound down to parietes or retained within body wall	One or two on VI, VII, VIII, and IX	One near the each spermatheca	None	Invisible	None	None

The new species is also fairly close to *Amynthascorticis* (Kinberg, 1867) by having medium to large size, spermathecal pores location and simple intestinal caeca (Table 2). However, the new species differs from *A.corticis* in pigmentation (yellowish brown dorsum and pale yellowish brown ventrum), first dorsal pore in 9/10, paired papillae within spermathecal pore region before setae in VIII or IX, male pores middle and round by 1/3C, two or more papillae near male pore, prostate glands in XIV–XXI with a large lumpy accessory gland, ampulla heart-shaped, diverticulum terminal 1/2 dilated into rod-shaped seminal chamber, paired large lumpy accessory glands on VIII or IX. Whereas *A.corticis* has a greenish brown dorsum and an unpigmented ventrum, first dorsal pore in 11/12, paired papilla before or after setae in some or all near spermathecal pores, male pores small and circular to transverse elliptical disc by 1/4C–1/3C, one or more papillae near male pore, prostate glands in XVII–XX without accessory glands, ampulla ovoid, diverticulum straight stalked, terminally dilated into a blunt ovoid seminal chamber, accessory glands stalked, coelomic, bound down to the parietes or retained within body wall.

Another similar species with four pairs of spermathecal pores in 5/6–8/9 is *Amynthasmaximus* Qiu & Dong, 2019. The two species share some similarities, such as body size, pigmentation, spermathecal pores, simple intestinal caeca (Table 2). However, *A.xiangtanensis* sp. nov. has first dorsal pore in 9/10, clitellum XIV–XVI, spermathecal pores, paired papillae before setae in VIII or IX, male pores without ridges, separated by 1/3 of body circumference, two or more papillae near male pore, prostate glands in XIV–XXI with a large lumpy accessory gland, spermathecae larger, ampulla heart-shaped, duct ~ 1/4 of ampulla, diverticulum terminal 1/2 dilated into rod-shaped seminal chamber, paired large lumpy accessory glands on VIII or IX. While *A.maximus* has first dorsal pore in 13/14, clitellum in 2/5XIV–XVI, fewer setae at VIII and XX, two pairs papillae after setae in VII and VIII, male pores surrounded by 3–4 circular ridges, separated by 2/5 of body circumference, paired papillae before setae medial of male pore, prostate glands in XVII–XIX with accessory glands invisible, spermathecae smaller, ampulla elongate-oval, duct 3/5 of ampulla, diverticulum terminal 2/5 dilated into a swollen, club-shaped seminal chamber, one or two stalked accessory glands on VI, VII, VIII and IX.

Regarding the pigmentation, clitellum, the position of spermathecal and male pores, simple intestinal caeca, and characteristics of the spermathecae, the new species is similar to *Amynthastortuosus* Qiu & Dong, 2019. However, the two species are distinguished by body size, the position of the first dorsal pore, setae number, the number and position of papillae within the spermathecal pore and male pore region, the characteristics and ventral distance of male pores, the position of prostate glands and the existence or nonexistence of accessory glands, and the characteristics of the diverticulum and accessory glands (Table 2).

In addition, the new species is somewhat similar to *Amynthasstricosus* Qiu & Sun, 2012 in the clitellum, the position of spermathecal and male pores, the ventral distance of male pores, and the simple intestinal caeca. Nevertheless, the two species are different in body size, pigmentation, the position of the first dorsal pore, setae number, the ventral distance of spermathecal pores and the existence or nonexistence of papillae, the characteristics of male pores, the number and position of papillae within the male pore region, the position of prostate glands and the existence or nonexistence of accessory glands, the characteristics of spermathecae, diverticulum and existence or nonexistence of accessory glands (Table 2).

#### ﻿ Amynthas taoyuanensis

Taxon classificationAnimaliaOligochaetaMegascolecidae

Qiu & Jin sp. nov.

21FE19A9-0A42-51EF-9FC6-64467170CB31

https://zoobank.org/99EAAAC1-F761-4082-A96E-645F864F631E

Fig. 3

##### Material examined.

***Holotype*.** • 1 clitellate (P1CJHUSH190517069 N8-01A), China, Hunan Province, Yongzhou City (25.30170°N, 111.63432°E), 201 m elevation, brown soil under weeds in dry farmland, 17 May 2019, JB Jiang, JL Li and BY Yin. ***Paratypes*.** 7 clitellates in total • 3 clitellates (P1CJHUSH190517069 N8-01B), China, Hunan Province, Yongzhou City (25.30170°N, 111.63432°E), 201 m elevation, brown soil under weeds in dry farmland, 17 May 2019, JB Jiang, JL Li and BY Yin • 1 clitellates (P1CJHUSH190526781 R8-04), China, Hunan Province, Liling City (27.63491°N, 113.54768°E), 55 m elevation, yellow soil under weeds in woodland, 26 May 2019, JB Jiang, JL Li and Y Wang • 2 clitellates (HU201601-03), China, Hunan Province, Changde City (28.70779°N, 111.38225°E), 66 m elevation, brown soil under moss and fern in farmland, 2 May 2016, JB Jiang, J Sun, Y Dong and Y Zheng • 1 clitellates (HU201602-04), China, Hunan Province, Changde City (28.65267°N, 111.34672°E), 69 m elevation, reddish brown soil under moss and fern in farmland, 2 May 2016, JB Jiang, J Sun, Y Dong and Y Zheng.

##### Diagnosis.

Size small to medium. Spermathecal pores in 5/6–8/9, separated by 1/3 of body circumference. Male pores in XVIII, separated by 1/3 of body circumference, each on the top of a slightly raised, circular porophore. Spermathecae four pairs in VI–IX, ampulla heart- or rod-shaped, duct is thick and ~ 1/3 of ampulla. Diverticulum is ~ 3/4 of main pouch (duct and ampulla together), terminal 3/4 dilated into bag-shaped chamber intestinal caeca are simple. Prostate glands are degenerated.

##### External characters.

**Figure 3.**
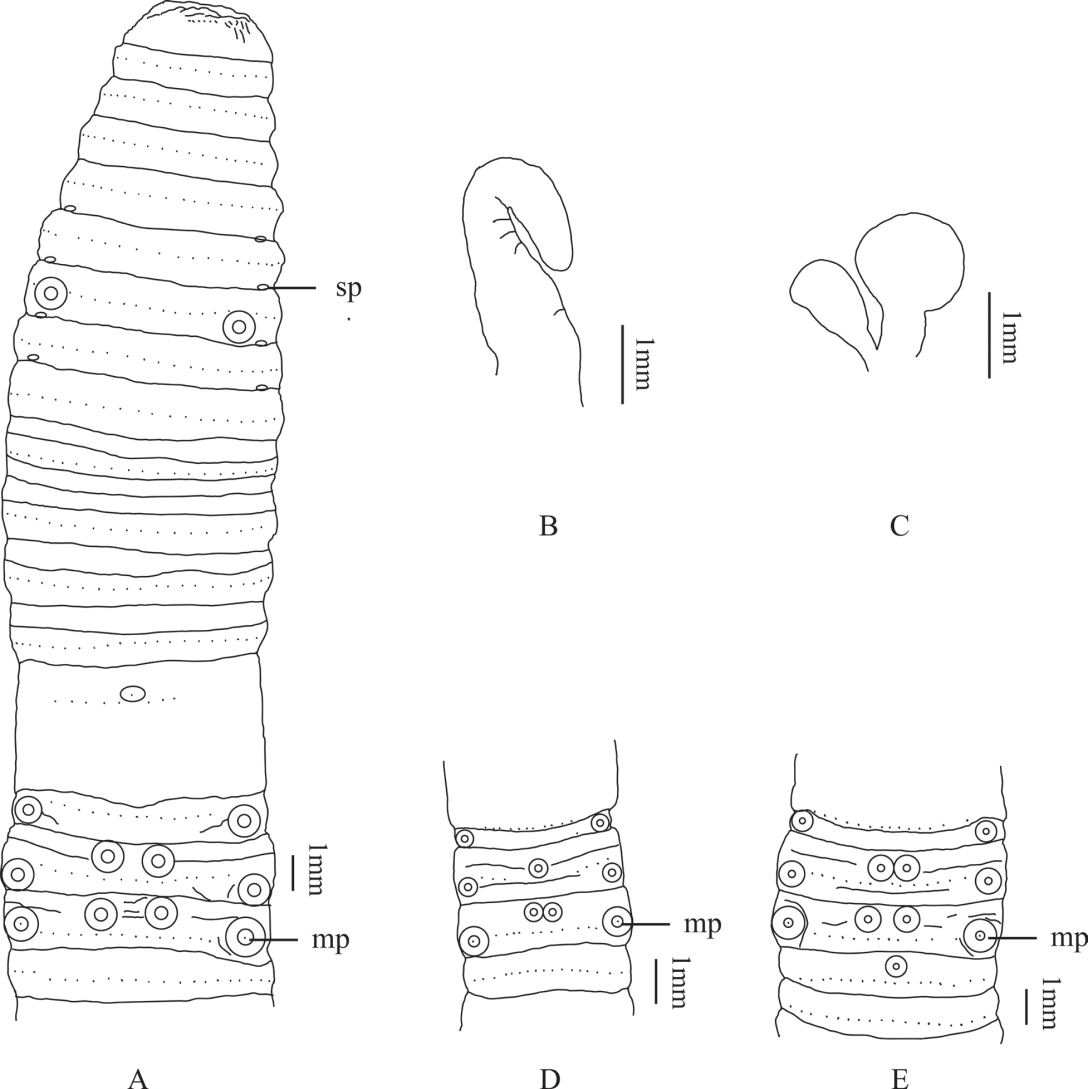
*A.taoyuanensis* sp. nov. **A** ventral view showing spermathecal pores, and male pores **B** intestinal caeca **C** spermathecae **D** male pores of paratypes (HU201601-03) **E** male pores of paratypes (HU201602-04). Abbreviations: sp, spermathecal pores; mp, male pores.

No pigmentation on dorsal and ventral. Dimensions 41–120 mm by 3.5–4.5 mm at clitellum, segments 49–112. Annulus present on X–XIII. The dorsal midline is clearly visible and green-brown. First dorsal pore of all examined individuals in 10/11. Prostomium 1/2 epilobous. Clitellum annular, grey, in XIV–2/3XVI, smooth, setae numbering 8 at XIV (P1CJHUSH190517069 N8-01 and HU201602-04), 11 at XV (HU201602-04), 13 at XVI (P1CJHUSH190517069 N8-01) or 14 at XVI (P1CJHUSH190526781 R8-04), dorsal pores visible on clitellum. Setae numbering 32–42 at III, 40–48 at V, 44–50 at VIII, 50–58 at XX, 56–62 at XXV; 13–14 between male pores; 18–19 (V), 18–21 (VI), 19–21 (VII) and 19–22 (VIII) between spermathecal pores; setal formula, aa = 1.0–1.4ab, zz = 1.0–1.4zy. Male pores one pair in XVIII, separated by 1/3 body circumference, each on the top of a slightly raised, circular porophore. Two pairs of circular medium-sized flat-topped papillae present after the setae ring in XVI and XVII, the interval is ~ 1/3 of body circumference. Two pairs of circular medium-sized flat-topped papillae present before the setae ring on XVII and XVIII, the interval is ~ 1/9 of body circumference (Fig. 3A). Sometimes a similar papilla presents in the center of XVII (specimen HU201601-03) (Fig. 3D). Sometimes another similar papilla presents in the center of XIX (specimen HU201602-04) (Fig. 3E). Female pore single in XIV, oval, milky white. Spermathecal pores four pairs in 5/6–8/9, ventral, small eye-like, milky white porophore in center, separated by 1/3 body circumference. A pair of oval medium-sized flat-topped papillae after the setae ring between the spermathecal pores in VII, the interval is ~ 1/4 of body circumference. Sometimes another pair of similar papillae present in the same position of VI (specimen HU201601-03 and HU201602-04). Sometimes another pair of similar papillae present in the same position of VIII (specimen P1CJHUSH190526781 R8-04).

##### Internal characters.

Septa 5/6–7/8 thick and muscular, 10/11–12/13 slightly thickened, 8/9 and 9/10 absent. Gizzard spherical, in IX–X. Intestine enlarged distinctly from XV. Intestinal caeca paired in XXVII, extending anteriorly to XXIV, simple, smooth on both sides (Fig. 3B). Four esophageal hearts in XI–XIII, well developed. Male sexual system holandric, testis sacs two pairs, in X and XI, well developed, left and right lobes separated on the ventral side. Seminal vesicles two pairs, extending in XI and XII, well developed, left and right lobes separated on the ventral side. Prostate glands degenerated, prostatic duct U-shaped inserting in XVIII, several small lumpy accessory glands in center of ventral XVI, XVII, and XVIII; the position is consistent with the position of the ventral mastoid on the body surface. Spermathecae four pairs in VI–IX, ampulla oval-shaped, ~ 0.6–1.8 mm long in holotype; ampulla duct is thick and ~ 1/2 of ampulla. Diverticulum is ~ 2/3 of main pouch (duct and ampulla together), terminal 3/4 dilated into bag-shaped chamber. Two pairs of large lumpy accessory glands present on the ventrum of VII and VIII (Fig. 3C). Sometimes pair of large lumpy accessory glands present on the ventrum of VII (specimen HU201601-03 and HU201602-04).

##### Etymology.

The species is named after its type locality.

##### Remarks.

*Amynthastaoyuanensis* sp. nov., with four pairs of spermathecal pores in 5/6–8/9, also belongs to the *Amynthascorticis* group. *Amynthastaoyuanensis* sp. nov. is close to *Amynthasxiangtanensis* sp. nov. in setae number, the position and characteristics of spermathecal pores and male pores, and the simple intestinal caeca. However, the differences between the two new species are body size, pigmentation, the position of the first dorsal pore, the position of clitellum, the position of papillae within spermathecal pores and male pore region, the existence or nonexistence of prostate glands, and the characteristics of the spermathecae and diverticulum (Table 2).

The new species is similar to *Amynthashomosetus* (Chen, 1938) in terms of body size, position and characteristics of spermathecal pores and male pores, and simple intestinal caeca. Whereas, the new species differs from *A.homosetus* in being unpigmented, the first dorsal pore in 10/11, clitellum in XIV–2/3XVI, 13 or 14 setae between male pores, the ventral distance of spermathecal pores is 1/3C, papillae paired after setae in VI, VII, or VIII, two paired or more papillae in XVI, XVII, XVIII, or XIX, prostate glands degenerated, spermathecae ~ 0.6–1.8 mm long, ampulla oval-shaped, duct is thick and ~ 1/2 of ampulla, diverticulum terminal 3/4 dilated into bag-shaped seminal chamber, two paired large lumpy accessory glands on VII or VIII. *Amynthashomosetus* is dark chocolate-colored anteriorly and grey on other parts of dorsum, grey on the ventrum, the first dorsal pore in 12/13, clitellum without setae in XIV–XVI, nine setae between male pores, the ventral distance of spermathecal pores is 1/4C, papillae invisible within spermathecal pores and male pore regions, prostate glands developed in XVI–XXI, spermathecae heart-shaped, diverticulum seminal chamber ovoid (Table 2).

*Amynthastaoyuanensis* sp. nov. is also close to *Amynthasstricosus* Qiu & Sun, 2012 by body size, pigmentation, setae number, the characteristics of spermathecal pores and male pores, the ventral distance of male pores, simple intestinal caeca. However, the new species is distinguished from *A.stricosus* by the first dorsal pore, the position and existence or nonexistence setae of clitellum, and the ventral distance of spermathecal pores; *A.taoyuanensis* sp. nov. has paired papillae after setae in VI, VII, or VIII, but *A.stricosus* has no papillae; in addition, *A.taoyuanensis* sp. nov. has two paired papillae or more in XVI, XVII, XVIII, or XIX, but *A.stricosus* has a single or paired after setae in XVII, XIX, and XX; prostate glands of *A.taoyuanensis* sp. nov. are degenerated with several lumpy accessory glands in XVI, XVII, and XVIII, while developed in XVI–XX with accessory glands invisible in *A.stricosus*; furthermore, spermathecae of *A.taoyuanensis* sp. nov. are ~ 0.6–1.8 mm long, ampulla oval-shaped, duct is thick and ~ 1/2 of ampulla, whereas ~ 1.6 mm long, ampulla heart-shaped, gradually slender duct as long as ampulla in *A.stricosus*; diverticulum of *A.taoyuanensis* sp. nov. is ~ 3/4 of main pouch, terminal 3/4 dilated into bag-shaped seminal chamber, but as long as main pouch, slender, terminal 2/5 dilated into band-shaped seminal chamber in *A.stricosus*; two paired large lumpy accessory glands on VII or VIII in *A.taoyuanensis* sp. nov., but no accessory glands in *A.stricosus* (Table 2).

According to body size, pigmentation, setae ventrally in clitellum, the position and characteristics of spermathecal pores and male pores, simple intestinal caeca, and the characteristics of spermathecae, *A.taoyuanensis* sp. nov. is somewhat similar to *Amynthasgenitalis* Qiu & Sun, 2012. However, the new species is characterized by the first dorsal in 10/11, clitellum in XIV–2/3 XVI, 19–22 setae between spermathecal pores (VIII), papillae paired within spermathecal pore region, two paired or more papillae in XVI, XVII, XVIII, or XIX, prostate glands degenerated with several lumpy accessory glands in XVI, XVII, and XVIII, diverticulum ~ 3/4 of main pouch, terminal 3/4 dilated into bag-shaped seminal chamber, two paired large lumpy accessory glands on VII or VIII; whereas *A.genitalis* has 8–11 setae between spermathecal pores (VIII), no papillae within spermathecal pore region, paired papillae before setae annulet in XVIII and XIX, after setae annulet in XVII and XVII, prostate glands developed with accessory glands invisible in XVII–XX, diverticulum longer than main pouch, slender, terminal 0.29 dilated into rod-shaped seminal chamber, no accessory glands (Table 2).

Another similar species with four pairs of spermathecal pores in 5/6–8/9 is *Amynthasrecavus* Yuan & Jiang, 2019. The two species share some similarities, such as the position of male pores and simple intestinal caeca. However, the two species are distinguished by body size, pigmentation, the first dorsal pore position, setae number, the ventral distance of spermathecal pores, existence or nonexistence of papillae within spermathecal pore region, the characteristics of male pores and papillae within male pore region, prostate glands, the characteristics of spermathecae and diverticulum, and the existence or nonexistence of accessory glands (Table 2).

#### ﻿ Amynthas xuanchengensis

Taxon classificationAnimaliaOligochaetaMegascolecidae

Jin & Li sp. nov.

5854E979-906F-5FDA-B066-56813555716C

https://zoobank.org/0CB4FE26-B76E-43AE-95F6-0A2B42139212

Fig. 4

##### Material examined.

***Holotype*.** • 1 clitellate (AH201517-06), China, Anhui Province, Xuancheng City (30.89694°N, 118.48889°E), 860 m elevation, black sandy soil under shrubbery in front of the house, 11 October 2015, Y Dong, Z Yuan, MS Chen and YL Wang. ***Paratype*.** • 1 clitellate (P1CJHUSH190521800 Q6-03), China, Hunan Province, Chenzhou City (26.16809°N, 113.05648°E), 79 m elevation, sandy soil under weeds beside roadways, 21 May 2019, JB Jiang, JL Li and Y Wang.

##### Diagnosis.

Size small. Spermathecal pores in 5/6 and 6/7, separated by 1/3 of body circumference. Male pores in XVIII, separated by 1/4 of body circumference, each on the top of a slightly raised, circular porophore. Spermathecae two pairs in VI and VII, ampulla heart-shaped, duct is thick and ~ 1/2 of ampulla. Diverticulum as long as main pouch (duct and ampulla together), terminal 1/2 dilated into ovoid-shaped seminal chamber. Intestinal caeca are simple. Prostate glands are developed.

##### External characters.

**Figure 4.**
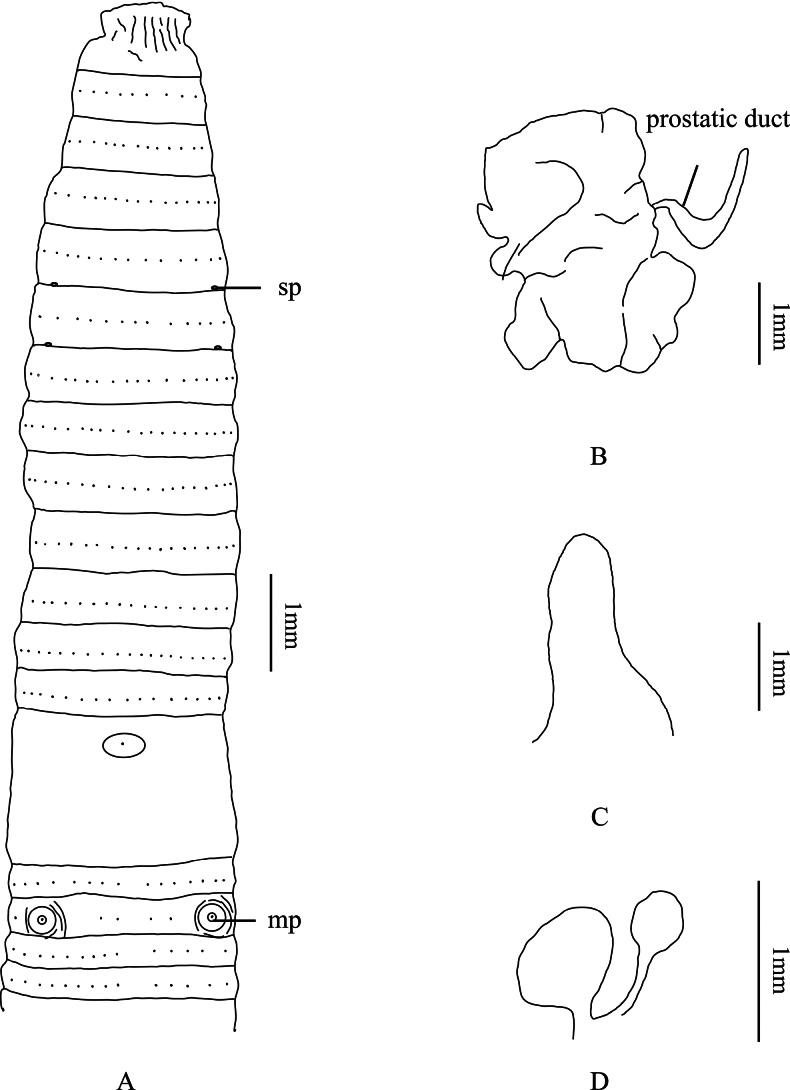
*A.xuanchengensis* sp. nov. **A** ventral view showing spermathecal pores, and male pores **B** prostate glands **C** intestinal caeca **D** spermathecae. Abbreviations: sp, spermathecal pores; mp, male pores.

No dorsal or ventral pigmentation. Dimensions 26–32 mm by 1.5–2.0 mm at clitellum, segments 60–65. The dorsal midline is not clearly visible. First dorsal pore of all examined individuals in 12/13. Prostomium 1/2 epilobous. Clitellum annular, taupe, in XIV– XVI, smooth, setae invisible externally. Setae numbering 32–40 at III, 36–44 at V, 40–48 at VIII, 50–54 at XX, 56–58 at XXV; 4 between male pores; setal formula, aa = 1.0–1.4ab, zz = 1.4–2.0zy. Male pores one pair in XVIII, separated by 1/4 body circumference, each on the top of a slightly raised, circular porophore (Fig. 4A). Female pore single in XIV, oval, milky white. Spermathecal pores two pairs in 5/6 and 6/7, ventral, not clearly, milky white porophore in center, separated by 1/3 body circumference.

**Figure 5.**
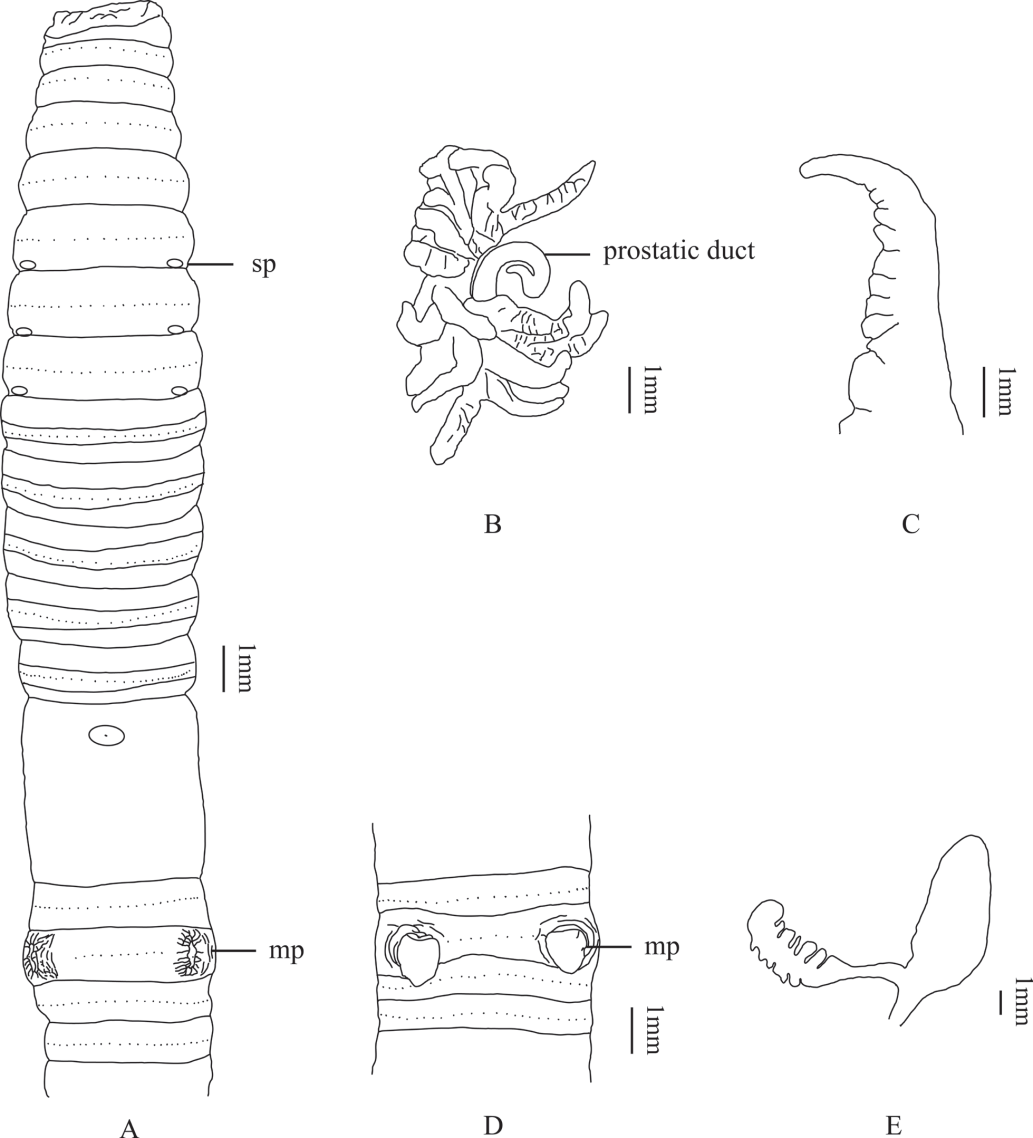
*M.donganensis* sp. nov. **A** ventral view showing spermathecal pores, and male pores **B** prostate glands **C** intestinal caeca **D** male pores of paratype (P1CJHUSH190511779 N9-03) **E** spermathecae. Abbreviations: sp, spermathecal pores; mp, male pores.

##### Internal characters.

Septa 5/6–7/8 thick and muscular, 10/11–12/13 slightly thickened, 8/9 and 9/10 absent. Gizzard spherical in IX–X. Intestine enlarged distinctly from XV. Intestinal caeca paired in XXVII, extending anteriorly to XXVI, simple, smooth on both sides (Fig. 4C). Four esophageal hearts in X–XIII, not well developed. Male sexual system holandric, testis sacs two pairs, in X and XI, well developed, left and right lobes separated on the ventral side. Seminal vesicles two pairs, extending in XI and XII, well developed, left and right lobes separated on the ventral side. Prostate glands well developed, inserting in XVIII and extending to XVI and XIX, coarsely lobate, prostatic duct U-shaped, slightly thicker at the distal part (Fig. 4B). No accessory glands observed. Spermathecae two pairs in VI–VII, ampulla heart-shaped, ~ 0.7 mm long in holotype; ampulla duct is thick and ~ 1/2 of ampulla. Diverticulum as long as main pouch (duct and ampulla together), terminal 1/2 dilated into ovoid-shaped seminal chamber. No accessory glands observed (Fig. 4D).

##### Etymology.

The species is named after its type locality.

##### Remarks.

*Amynthasxuanchengensis* sp. nov., with two pairs of spermathecal pores in 5/6 and 6/7, can be assigned to *Amynthasmorrisi* group (Sims and Easton 1972) with 55 species included in this group (Sun et al. 2009, 2015, 2021; Shen et al. 2014; Jiang et al. 2015; Hong 2017), such as *Amynthasnanulus* (Chen & Yang, 1975) (Chen et al. 1975), *Amynthasparvus* (Chen & Hsu, 1977), *Amynthaspiagolensis* Hong & James, 2001, *Amynthasdiaoluomontis* Qiu & Sun, 2009, *Amynthasendophilus* Zhao & Qiu, 2013. *Amynthasxuanchengensis* sp. nov. is very similar to *Amynthasendophilus* Zhao & Qiu, 2013 by lacking pigmentation, first dorsal pore in 12/13, clitellum in XIV–XVI, the position of spermathecal pores and male pores, no papillae within spermathecal pore and male pore regions, simple intestinal caeca. However, the new species differs from *A.endophilus* by smaller body size and fewer setae. And beyond that, the ventral distance of spermathecal pores and male pores is 1/3C and 1/4C in *A.xuanchengensis* sp. nov., but 2/5C and 1/3C in *A.endophilus*; spermathecal pores of *A.xuanchengensis* sp. nov. are inconspicuous, but obvious in *A.endophilus*; male pores of *A.xuanchengensis* sp. nov. not surrounded by folds, but *A.endophilus* surrounded by four folds; prostate glands in XVI–XIX in *A.xuanchengensis* sp. nov., but XVII–XXI in *A.endophilus*; spermathecae ~ 0.7 mm long, ampulla heart-shaped, duct is thick and ~ 1/2 of ampulla of *A.xuanchengensis* sp. nov., while spermathecae longer, ampulla elongated ovoid, duct a little shorter than ampulla in *A.endophilus*; diverticulum as long as main pouch in *A.xuanchengensis* sp. nov., nevertheless shorter than main pouch in *A.endophilus* (Table 3).

**Table 3.** A comparison of characters of *A.xuanchengensis* sp. nov. and similar species of the *Amynthasmorrisi* group. Abbreviations: sp, spermathecal pores, mp, male pores.

Character	*A.xuanchengensis*	*A.endophilus*	*A.fucatus*	*A.infuscuatus*	*A.zonarius*	*A.baikmudongensis*
Body size (mm)	26–32*1.5–2.0	96*3	137*4.0	60–78*1.4–1.6	52–103*1.6–3.1	75*4.3
Pigment dorsum	None	None	Dark red brown to pale	Purple to pale brown	None	None
Pigment ventrum	None	None	Pale red brown to pale	None	None	-
Setae numbering	32–40/III, 40–48/VIII, 50–54/XX	56/III, 72/V, 84/VIII	44/III, 60/VIII, 40/XX	46–48/III, 49–52/VIII, 42–46/XX	38–56/III, 46–52/VIII, 34–54/XX	24/VII, 53/XX
Setae number between mp	4 (XVIII)	2 (XVIII)	8 (XVIII)	8–10 (XVIII)	6–12 (XVIII)	7 (XVIII)
Spermathecal pores	Inconspicuous, 1/3C	Obvious, 2/5C	Obvious, 2/5C	Obvious, 1/3C	Inconspicuous, 2/5C	Inconspicuous
Male pores	1/4C	Surrounded by folds, 1/3C	Surrounded by folds, 2/5C	Surrounded by folds, 1/3C	Surrounded by folds, 1/3C	Large circular raised pads in XVIII
Papillae within mp region	None	None	Two in the inner side of male pore., another paired in XVIII	Two in the inner side of male pore	One or two in the inner side of male pore, sometimes another two on XVIII	None
Prostate glands	Developed	Developed	Well developed	Developed	Developed	Underdeveloped
Diverticulum	As long as main pouch, terminal 1/2 dilated into ovoid-shaped seminal chamber	4/5 of main pouch, terminal 3/5 dilated into zonal seminal chamber	1/3 of main pouch, terminal 2/5 dilated into virgulate seminal chamber	Slightly longer than main pouch, terminal 1/3 dilated into swollen seminal chamber	Long as main pouch, terminal 1/2 dilated into band-shaped seminal chamber	1/2 of main pouch, terminal 1/2 dilated into club-shaped seminal chamber

Another similar species with two pairs of spermathecal pores in 5/6 and 6/7 is *Amynthasfucatus* Zhao & Qiu, 2013. The two species share some similarities, such as clitellum in XIV–XVI, the position of spermathecal pores and male pores, invisible papillae within spermathecal pore region, and simple intestinal caeca. By contrast, the new species and *A.fucatus* can be separated on the basis of smaller body size and fewer setae at VIII. In addition, *A.xuanchengensis* sp. nov. is unpigmented, but dark red brown before clitellum and pale after clitellum in dorsum, pale red brown before clitellum and pale after clitellum in ventrum in *A.fucatus*; the first dorsal pore of *A.xuanchengensis* sp. nov. in 12/13, but 11/12 in *A.fucatus*; the ventral distance of spermathecal pores and male pores are 1/3C and 1/4C in *A.xuanchengensis* sp. nov., but 2/5C and 2/5C in *A.fucatus*; spermathecal pores of *A.xuanchengensis* sp. nov. are inconspicuous, but obvious in *A.fucatus*; male pores of *A.xuanchengensis* sp. nov. not surrounded by folds, but *A.fucatus* surrounded by three to five folds; invisible papillae within male pore region of *A.xuanchengensis* sp. nov., whereas two papillae on the inner side of male pore, another paired in XVIII in *A.fucatus*; prostate glands in XVI–XIX of *A.xuanchengensis* sp. nov., while XV–XXI with accessory glands invisible in *A.fucatus*; spermathecae ~ 0.7 mm long, ampulla heart-shaped in *A.xuanchengensis* sp. nov., nevertheless spermathecae longer, ampulla elongated ovoid in *A.fucatus*; diverticulum is as long as main pouch, terminal 1/2 dilated into ovoid-shaped seminal chamber in *A.xuanchengensis* sp. nov., but diverticulum ~ 1/3 of main pouch, straight, terminal 3/4 dilated into virgulate seminal chamber in *A.fucatus* (Table 3).

*Amynthasxuanchengensis* sp. nov. appears to be closely related to *A.infuscuatus* Jiang & Sun, 2015 in the combined characters of ventrum pigmentation, first dorsal pore in 12/13, clitellum in XIV–XVI, the position and characteristics of spermathecal pores, invisible papillae within spermathecal pore region, the position of male pores, simple intestinal caeca, and ampulla heart-shaped. Conversely, they still have some differences, such as body size, dorsum pigmentation, and setae number. *Amynthasxuanchengensis* sp. nov. smaller, without pigment on dorsum, but purple before VIII; pale brown after VIII in *A.infuscuatus*; four setae between male pores of *A.xuanchengensis* sp. nov., but 8–10 setae in *A.infuscuatus*; the ventral distance of male pores is 1/4C in *A.xuanchengensis* sp. nov., but 1/3C in *A.infuscuatus*; spermathecal pores of *A.xuanchengensis* sp. nov. are inconspicuous, but obvious in *A.infuscuatus*; male pores of *A.xuanchengensis* sp. nov. surrounded by no folds, but *A.infuscuatus* surrounded by three or four folds; invisible papillae within male pore region of *A.xuanchengensis* sp. nov., whereas two papillae on the inner side of male pore in *A.infuscuatus*; prostate glands in XVI–XIX of *A.xuanchengensis* sp. nov., while XVI–1/2XX with accessory glands invisible in *A.infuscuatus*; spermathecae ~ 0.7 mm long, duct is thick and ~ 1/2 of ampulla in *A.xuanchengensis* sp. nov., but spermathecae longer, duct slender, twice as long as ampulla in *A.infuscuatus*; diverticulum is as long as main pouch, terminal 1/2 dilated into ovoid-shaped seminal chamber in *A.xuanchengensis* sp. nov., but diverticulum longer than main pouch, slender, terminal 1/3 dilated into swollen seminal chamber in *A.infuscuatus* (Table 3).

*Amynthasxuanchengensis* sp. nov. and *Amynthaszonarius* Sun & Qiu, 2015 share some common characters in pigmentation, first dorsal pore in 12/13, clitellum in XIV–XVI, the characteristics of spermathecal pores, invisible papillae within spermathecal pore region, the position of male pores, simple intestinal caeca. In contrast, *A.xuanchengensis* sp. nov. differs from *A.zonarius* in smaller body size and fewer setae. Additionally, the ventral distance of spermathecal pore and male pores are 1/3C and 1/4C in *A.xuanchengensis* sp. nov., but 2/5C and 1/3C in *A.zonarius*; male pores of *A.xuanchengensis* sp. nov. surrounded by no folds, but *A.zonarius* surrounded by five folds; invisible papillae within male pore region of *A.xuanchengensis* sp. nov. whereas one or two papillae on the inner side of male pore, sometimes another two on XVIII in *A.zonarius*; prostate glands in XVI–XIX in *A.xuanchengensis* sp. nov., while XVI–1/2XX with accessory glands invisible in *A.zonarius*; spermathecae ~ 0.7 mm long, ampulla heart-shaped, duct is thick and ~ 1/2 of ampulla in *A.xuanchengensis* sp. nov., whereas spermathecae longer, ampulla ovoid, duct as long as ampulla in *A.zonarius*; ovoid-shaped seminal chamber in *A.xuanchengensis* sp. nov., but band-shaped seminal chamber in *A.zonarius* (Table 3).

In terms of pigmentation, clitellum, setae number, the positions of spermathecal pores and male pores, inconspicuous spermathecal pores, invisible papillae within spermathecal pore and male pore regions, simple intestinal caeca. *Amynthasxuanchengensis* sp. nov. is somewhat similar to *Amynthasbaikmudongensis* Hong, 2017. On the contrary, the new species is easily distinguished from *A.baikmudongensis* by body size, prostate glands, the characteristics of male pores, spermathecae, and diverticulum (Table 3).

### ﻿Genus *Metaphire* Sims & Easton, 1972

#### ﻿ Metaphire donganensis

Taxon classificationAnimaliaOligochaetaMegascolecidae

Jin & Jiang sp. nov.

8E1C41F9-35AA-5322-BC26-D62CB1A52E22

https://zoobank.org/25F88443-9F42-49B6-AC05-114236A5D710

Fig. 5

##### Material examined.

***Holotype*.** • 1 clitellate (HU201613-01A), China, Hunan Province, Dongan City (26.35499°N, 111.19531°E), 172 m elevation, brown soil under vegetable field in farmland, 4 May 2016, JB Jiang, J Sun, Y Dong, and Y Zheng. ***Paratypes*.** 13 clitellates in total • 1 clitellate (HU201613-01B), China, Hunan Province, Dongan City (26.35499°N, 111.19531°E), 172 m elevation, brown soil under vegetable field in farmland, 4 May 2016, JB Jiang, J Sun, Y Dong and Y Zheng • 2 clitellates (P1CJHUSH190511779 N9-03), China, Hunan Province, Liuyang City (28.32795°N, 113.52008°E), 112 m elevation, red soil under vegetable in vegetable garden, 11 May 2019, Y Dong, YF Qin and YZ Wu • 7 clitellates (P1CJHUSH190512778 N11-01), China, Hunan Province, Yueyang City (28.91995°N, 113.70132°E), 179 m elevation, brown soil under rape in rape field, 12 May 2019, Y Dong, YF Qin and YZ Wu • 1 clitellate (P1CJHUSH190512096 Q3-04), China, Hunan Province, Linxiang City (29.33550°N, 113.40176°E), 51 m elevation, brown sandy soil under shrub in grove, 12 May 2019, Y Dong, YF Qin and YZ Wu • 2 clitellates (P1CJHUSH190518092 Q6-01), China, Hunan Province, Yuanjiang City (29.04369°N, 112.29798°E), 48 m elevation, brown soil under litter next to the house, 18 May 2019, Y Dong, YF Qin and YK Li.

##### Diagnosis.

Size medium to large. Spermathecal pores in 6/7–8/9, separated by 1/3 of body circumference. Male pores in XVIII, separated by 1/3 of body circumference, each on the bottom center of the longitudinal copulatory chamber. Sometimes the copulatory chamber eversion is ridged. Spermathecae three pairs in VII–IX, ampulla heart- or rod-shaped, duct thick and as long as ampulla. Diverticulum as long as main pouch (duct and ampulla together), slender and straight at proximal part, terminal 1/2 dilated into twisted in zigzag fashion. Intestinal caeca are simple. Prostate glands are well developed.

##### External characters.

Pale brown dorsal and ventral pigmentation. Dimensions 72–159 mm by 4.6–7.0 mm at clitellum, segments 78–111. Annulus present on IX–XVIII. The dorsal midline is clearly visible and purplish brown. First dorsal pore of all examined individuals in 12/13. Prostomium 1/2 epilobous. Clitellum annular, pale taupe, in XIV– XVI, smooth, setae invisible externally. Setae numbering 20–34 at III, 20–46 at V, 28–58 at VIII, 52–66 at XX, 58–70 at XXV; 8–13 between male pores; 14–17 (VI), 16–20 (VII) and 17–21 (VIII) between spermathecal pores; setal formula, aa = 1.0–1.6ab, zz = 1.2–1.8zy. Male pores one pair in XVIII, separated by 1/3 body circumference, each on the bottom center of the longitudinal copulatory chamber, multiple radioactive folds on the outer edge (Fig. 5A). Sometimes the copulatory chamber eversion is ridged (specimen P1CJHUSH190511779 N9-03, P1CJHUSH190512096 Q3-04 and P1CJHUSH190518092 Q6-01) (Fig. 5D). Female pore single in XIV, oval, milky white. Spermathecal pores three pairs in 6/7–8/9, ventral, large eye-like, milky white porophore in center, separated by 1/3 body circumference.

##### Internal characters.

Septa 5/6–7/8 thick and muscular, 10/11–12/13 slightly thickened, 8/9 and 9/10 absent. Gizzard spherical, in IX–X. Intestine enlarged distinctly from XV. Intestinal caeca paired in XXVII, extending anteriorly to XXIII, simple, smooth dorsal margin, weakly constricted on ventral margin (Fig. 5C). Four esophageal hearts in X–XIII, the latter three are more developed than the first pair. Male sexual system holandric, testis sacs two pairs, in X and XI, well developed, left and right lobes connected on the ventral side. Seminal vesicles two pairs, extending in XI and XII, well developed, left and right lobes separated on the ventral side. Prostate glands well developed, inserting in XVIII and extending to XVI and XXII, strip lobate, prostatic duct U-shaped, slightly thicker at the distal part (Fig. 5B). No accessory glands observed. Spermathecae three pairs in VII–IX, ampulla heart- or rod-shaped, ~ 3.2–8.0 mm long in holotype; ampulla duct is swollen and as long as ampulla. Diverticulum as long as main pouch (duct and ampulla together), slender and straight at proximal part, terminal 1/2 dilated into twisted in zigzag fashion. No accessory glands observed (Fig. 5E).

##### Etymology.

The species is named after its type locality.

##### Remarks.

*Metaphiredonganensis* sp. nov. with three pairs spermathecal pores in 6/7–8/9, keys to the *Metaphirehoulleti* group, which includes 44 species (Sims and Easton 1972; Feng 1984; Feng and Ma 1987; Qiu and Zhong 1993; Sun et al. 2018). *Metaphirevulgarisagricola* (Chen, 1930) is slightly akin to the new species in the respects of body size, first dorsal pore in 12/13, clitellum in XIV–XVI, setae number, the position and characteristics of spermathecal pores, simple intestinal caeca, and the characteristics of diverticulum. Instead, the difference between the two species is in the pigmentation, pale brown in *M.donganensis* sp. nov., earthy yellow in *M.vulgarisagricola* (Feng, 1981). Further, the ventral distance of spermathecal pore and male pores are 1/3C and 1/3 C in *M.donganensis* sp. nov., but 1/4C and 1/4C in *M.vulgarisagricola*; no papillae within spermathecal pore region of *M.donganensis* sp. nov., but paired papillae in VII of *M.vulgarisagricola*; no papillae within male pore region of *M.donganensis* sp. nov., while paired papillae on the inner side of male pore in *M.vulgarisagricola*; prostate glands well developed in XVI–XXII of *M.donganensis* sp. nov., nevertheless in XVII–XX with accessory glands invisible in *M.vulgarisagricola*; spermathecae ~ 3.2–8.0 mm long, ampulla heart- or rod-shaped, duct swollen, as long as ampulla of *M.donganensis* sp. nov., while spermathecae ~ 4.5 mm long, ampulla pear-shaped, ampulla duct 1/2 of ampulla in *M.vulgarisagricola*; no accessory glands in *M.donganensis* sp. nov., while paired accessory glands in the VIII of *M.vulgarisagricola* (Table 4).

**Table 4.** A comparison of characters of *M.donganensis* sp. nov. and similar species of the *Metaphirehoulleti* group. Abbreviations: sp, spermathecal pores, mp, male pores.

Character	*M.donganensis*	*M.vulgarisagricola*	*M.tschiliensislanzhouensis*	*M.viridis*	*M.ptychosiphona*	*M.sanmingensis*
Body size (mm)	72–159*4.6–7.0	118*6.0	245–310*6–7	192–230*9.5–10	196–295*6.0–9.0	55–113* 4–5.5
Pigment dorsum	Pale brown	Earthy yellow	Earthy yellow	Dark green	Grey-brown	Pale brown to brown
Pigment ventrum	Pale brown	Earthy yellow	Earthy yellow	Pale green	Grey-brown	None
First dorsal pore	12/13	12/13	12/13	12/13	11/12	11/12, 12/13 or 13/14
Setae numbering	20–34/III, 28–58/VIII, 52–66/XX	29/III, 54/VIII, 62/XXV	32–40/III, 46–55/VIII	47–50/III, 64–67/VIII, 92–95/XXV	57–61III, 64–79/VIII, 87–117/XX	16–24/III, 33–40/VIII, 44–48/XX
Setae number between sp	17–21 (VIII)	-	16–24 (VIII)	-	28–34 (VIII)	12 (VIII)
Setae number between mp	8–13 (XVIII)	-	8–14 (XVIII)	-	15–27 (XVIII)	8–9 (XVIII)
Ventral distance of sp	1/3C	1/4C	1/3C	1/2C	2/5C	1/3C
Papillae within sp region	None	Paired in VII	None	Paired in VII, VIII, and IX	None	Two in VII and VIII, or extra two paired in VIII
Ventral distance of mp	1/3C	1/4C	1/3C	1/2C	1/3C	1/3C
Papillae within mp region	None	Paired in the inner side of male pore	One in pouches	Four in pouches	None	Three in the inner side of male pores, extra three in XVIII
Prostate glands	Well developed	Developed	Underdeveloped with accessory gland	Developed with accessory gland	Well developed	Developed
Diverticulum	As long as main pouch, terminal 1/2 dilated into zigzag fashion	Shorter, terminal 2/3 dilated into twisted zigzag fashion	As long as main pouch, terminal 2/3 dilated into zigzag fashion	Longer, terminal 1/2 dilated into zigzag fashion	Shorter, terminal 0.6 dilated into zigzag fashion	Shorter, terminal dilated into rod-shaped seminal chamber
Accessory glands	None	Paired in VIII	None	Paired in VII, VIII, and IX	None	Invisible

We compare the new species to *Metaphiretschiliensislanzhouensis* (Feng, 1984), which has three pairs of spermathecal pores in 6/7–8/9. They share several common characters in the first dorsal pore position, clitellum in XIV–XVI, setae number, the position of spermathecal pores, the position and characteristics of male pores, no papillae within spermathecal pore region, simple intestinal caeca, and the characteristics of the diverticulum. Quite the contrary, the new species is smaller than *M.tschiliensislanzhouensis*. Beyond that, coloration is pale brown in *M.donganensis* sp. nov., earthy yellow in *M.tschiliensislanzhouensis*; spermathecal pores are obvious in *M.donganensis* sp. nov., but inconspicuous in *M.tschiliensislanzhouensis*; no papillae within male pore region of *M.donganensis* sp. nov., but one in pouch of *M.tschiliensislanzhouensis*; prostate glands well developed in XVI–XXII of *M.donganensis* sp. nov., while underdeveloped in XVII–XIX with a large lumpy accessory gland in *M.tschiliensislanzhouensis*; spermathecae ~ 3.2–8.0 mm long, ampulla heart- or rod-shaped of *M.donganensis* sp. nov., but spermathecae ~ 4 mm long, ampulla spherical-shaped in *M.tschiliensislanzhouensis* (Table 4).

Considering the three pairs of spermathecal pores in 6/7–8/9, we compared the new species with *Metaphireviridis* Feng & Ma, 1987. Both have first dorsal pores in 12/13, clitellum in XIV–XVI, the same position and characteristics of spermathecal pores, simple intestinal caeca, and the same characteristics of diverticulum. However, the new species differs from *M.viridis* in many respects. *Metaphiredonganensis* sp. nov. is smaller than *M.viridis* with pale brown coloration; setae number of *M.donganensis* sp. nov. is less than *M.viridis*; the ventral distance of spermathecal pore and male pores are 1/3C and 1/3 C in *M.donganensis* sp. nov., but 1/2C and 1/2C in *M.viridis*; no papillae within spermathecal pore region of *M.donganensis* sp. nov., while paired in VII, VIII, and IX of *M.viridis*; no papillae within male pore region of *M.donganensis* sp. nov., but four in pouches in *M.viridis*; prostate glands in XVI–XXII of *M.donganensis* sp. nov., while XV–XX or XVII–XIX with a lumpy accessory gland in *M.viridis*; no accessory glands in *M.donganensis* sp. nov., whereas paired in the VII, VIII and IX of *M.viridis*; spermathecae ~ 3.2–8.0 mm long, ampulla heart- or rod-shaped of *M.donganensis* sp. nov., nevertheless spermathecae ~ 4 mm long, ampulla spherical- or oval-shaped in *M.viridis*; no accessory glands of *M.donganensis* sp. nov., while paired in VII, VIII, and IX of *M.viridis* (Table 4).

We also compare the new species with *Metaphireptychosiphona* Qiu & Zhong, 1993 on the basis of the position and characteristics of male pores, no papillae within spermathecal pore and male pore regions, simple intestinal caeca, and the same characteristics of the diverticulum. Nevertheless, the new species is smaller than *M.ptychosiphona*. In addition, pale brown in *M.donganensis* sp. nov., but grey brown in *M.ptychosiphona*; first dorsal pore in 12/13 of *M.donganensis* sp. nov., while in 11/12 of *M.ptychosiphona*; setae number of *M.donganensis* sp. nov. less than *M.ptychosiphona*; the ventral distance of spermathecal pore is 1/3C in *M.donganensis* sp. nov., but 2/5C in *M.ptychosiphona*; spermathecal pores obvious in *M.donganensis* sp. nov., but inconspicuous in *M.ptychosiphona*; prostate glands in XVI–XXII of *M.donganensis* sp. nov., while XVI–XVIII or XVII–XIX in *M.ptychosiphona*; and spermathecae ~ 3.2–8.0 mm long, ampulla heart- or rod-shaped, duct swollen, as long as ampulla in *M.donganensis* sp. nov., nevertheless spermathecae shorter, ampulla rod-shaped, duct shorter than ampulla in *M.ptychosiphona* (Table 4).

The new species is also close to *Metaphiresanmingensis* Sun & Jiang, 2018 in body size, clitellum in XIV–XVI, the position of spermathecal pores and male pores, simple intestinal caeca, prostate glands, and the characteristics of the ampulla and diverticulum. However, the new species is easily distinguished from *M.sanmingensis* in pigmentation, first dorsal pore position, setae number, papillae within spermathecal pore region, and the characteristics of male pores and papillae within male pore region (Table 4).

### ﻿Molecular results

The COI mitochondrial DNA gene, considered as a barcode for earthworm identification (Huang et al. 2007; Novo et al. 2010), is an effective complement to morphological analyses. Comparisons of COI gene sequences among *A.xiangtanensis* sp. nov., *A.taoyuanensis* sp. nov., *A.xuanchengensis* sp. nov. *M.donganensis* sp. nov., *A.corticis*, *A.maximus*, *A.tortuosus*, *A.stricosus*, *A.recavus*, *A.endophilus*, *A.fucatus*, *A.zonarius* and *M.sanmingensis* yielded high pairwise distances (Table 5). According to Chang and James (2011), values above 10–15%, most probably indicate different species. It is clear that the new species and other species in Table 5 have large genetic divergences. In general, pairwise distances between the four new species and the other group species are greater than 17.17%. Together with the different morphological characteristics of each, we can conclude that *A.xiangtanensis* sp. nov., *A.taoyuanensis* sp. nov., *A.xuanchengensis* sp. nov., and *M.donganensis* sp. nov. are different from previously described species and each other.

**Table 5.** Percentage of pairwise distances of COI genes between 13 species.

	S1 HT	S1 PT	S1 PT	S2 HT	S2 PT	S3 PT	S4 PT	S4 PT	S4 PT	*A.corticis*	*A.maximus*	*A.tortuosus*	*A.stricosus*	*A.recavus*	*A.endophilus*	*A.fucatus*	*A.zonarius*	*M.sanmingensis*
S1 HT																		
S1 PT	0.00%																	
S1 PT	0.16%	0.16%																
S2 HT	21.78%	21.78%	21.55%															
S2 PT	21.78%	21.78%	21.55%	0.00%														
S3 PT	21.32%	21.32%	21.10%	17.25%	17.25%													
S4 PT	22.53%	22.53%	22.29%	18.81%	18.81%	20.64%												
S4 PT	22.29%	22.29%	22.06%	18.60%	18.60%	20.42%	0.16%											
S4 PT	22.27%	22.27%	22.04%	18.82%	18.82%	19.95%	5.62%	5.44%										
*A.corticis*	23.16%	23.16%	22.92%	20.83%	20.79%	23.25%	20.68%	20.45%	19.55%									
*A.maximus*	21.05%	21.05%	20.79%	19.13%	19.09%	18.25%	18.16%	17.91%	18.38%	15.54%								
*A.tortuosus*	19.78%	19.78%	19.55%	18.75%	18.71%	18.78%	17.05%	17.26%	17.25%	20.18%	19.18%							
*A.stricosus*	18.06%	18.06%	17.85%	16.53%	16.50%	16.62%	17.16%	16.95%	16.31%	16.91%	15.31%	14.18%						
*A.recavus*	18.54%	18.54%	18.32%	15.90%	15.87%	19.85%	15.08%	14.88%	15.93%	19.58%	19.57%	16.12%	14.69%					
*A.endophilus*	17.17%	17.17%	16.96%	17.58%	17.54%	17.61%	18.99%	18.77%	18.37%	18.29%	16.59%	17.68%	15.04%	17.32%				
*A.fucatus*	22.48%	22.48%	22.07%	21.67%	21.67%	23.34%	19.67%	19.29%	21.24%	17.82%	20.52%	18.22%	20.22%	18.67%	18.22%			
*A.zonarius*	18.04%	18.04%	17.82%	16.00%	15.97%	16.79%	19.05%	19.27%	21.04%	18.43%	16.02%	15.61%	14.38%	15.72%	13.66%	18.73%		
*M.sanmingensis*	20.81%	20.81%	20.57%	19.96%	19.92%	19.24%	19.30%	19.06%	20.00%	15.30%	19.69%	17.71%	15.00%	17.96%	18.58%	21.54%	15.88%	

Notes: S1 represent *A.xiangtanensis* sp. nov., S2 represent *A.taoyuanensis* sp. nov., S3 represent *A.xuanchengensis* sp. nov., S4 represent *M.donganensis* sp. nov.; Abbreviations: HT holotype, PT paratype.

## Supplementary Material

XML Treatment for Amynthas xiangtanensis

XML Treatment for Amynthas taoyuanensis

XML Treatment for Amynthas xuanchengensis

XML Treatment for Metaphire donganensis

## ﻿Additional information

### Author ORCIDs

Qing Jin https://orcid.org/0000-0001-7731-3150

AuthorName https://orcid.org/0000-0000-0000-0000

AuthorName https://orcid.org/0000-0000-0000-0000

### Data availability

All of the data that support the findings of this study are available in the main text.
